# Food security, diet quality, nutritional knowledge, and attitudes towards research in adults with heart failure during the COVID‐19 pandemic

**DOI:** 10.1002/clc.23761

**Published:** 2022-02-02

**Authors:** Andrew P. Ambrosy, Umar I. Malik, Thomas K. Leong, Amanda R. Allen, Sue Hee Sung, Alan S. Go, Healthy Eating as a Means for Active Living in Heart Failure (HEAL‐HF) Study

**Affiliations:** ^1^ Department of Cardiology Kaiser Permanente San Francisco Medical Center San Francisco California USA; ^2^ Division of Research Kaiser Permanente Northern California Oakland California USA; ^3^ Department of Health Systems Science Kaiser Permanente Bernard J. Tyson School of Medicine Pasadena California USA; ^4^ Departments of Epidemiology, Biostatistics and Medicine University of California, San Francisco San Francisco California USA; ^5^ Department of Medicine Stanford University Palo Alto California USA

**Keywords:** diet, food insecurity, heart failure, nutrition, outcomes

## Abstract

**Background:**

The impact of the novel coronavirus disease 2019 (COVID‐19) pandemic on diet and nutrition among older adults with chronic medical conditions have not been well‐described.

**Methods:**

We conducted a survey addressing (1) food access, (2) diet quality and composition, (3) nutritional understanding, and (4) attitudes towards research among adults with heart failure (HF) within an integrated health system. Adults (≥18 years) with diagnosed HF and at least one prior hospitalization for HF within the last 12 months were approached to complete the survey electronically or by mail. Outcomes included all‐cause and HF‐specific hospitalizations and all‐cause death was ascertained via the electronic health record.

**Results:**

Among 1212 survey respondents (32.5% of eligible patients) between May 18, 2020 and September 30, 2020, mean ± *SD* age was 77.9 ± 11.4 years, 50.1% were women, and median (25th–75th) left ventricular ejection fraction was 55% (40%–60%). Overall, 15.1% of respondents were food insecure, and only 65% of participants answered correctly more than half of the items assessing nutritional knowledge. Although most respondents were willing to participate in future research, that number largely declined for studies requiring blood draws (32.2%), study medication (14.4%), and/or behavior change (27.1%). Food security, diet quality, and nutritional knowledge were not independently associated with outcomes at 90 or 180 days.

**Conclusion:**

In a cohort of older adults with HF and multiple comorbidities, a significant proportion reported issues with food access, diet quality, and nutritional knowledge during the COVID‐19 pandemic. Future research should evaluate interventions targeting these domains in at‐risk individuals.

## Key points

- Upwards of 15% of respondents screened positive for food insecurity, and only approximately 65% of respondents answered more than half of the items correctly on a questionnaire assessing nutritional understanding.
- Although the majority of respondents indicated that would be willing to consider participating in future research, that proportion declined markedly for studies requiring blood draws, study drugs, and/or behavior change.
- None of the functional domains assessed by this survey were independently associated with clinical outcomes in our cohort.

## 1 INTRODUCTION

More than 1 million adult Americans are hospitalized for heart failure (HF) annually, accounting for 6.5 million hospital days and the majority of the approximately $40 billion spent each year on HF‐related care.^1^, ^2^ In addition, while per capita hospitalization rates may be beginning to decline, postdischarge readmission rates and mortality remain unacceptably high nationally.^3^ Thus, understanding the precipitants contributing to hospitalizations and readmissions for HF, particularly those that are potentially avoidable, may facilitate more effective HF disease management. Several risk factors have been identified including arrhythmias, myocardial ischemia, respiratory infections, uncontrolled hypertension, nonadherence to medications, and/or dietary indiscretion.^4^, ^5^, ^6^, ^7^, ^8^

The latter is particularly relevant as a previous study in adults admitted for worsening HF estimated that nonadherence with HF‐specific dietary recommendations potentially contributed to >5% of hospitalizations.^9^ Interestingly, a “heart‐healthy” diet, particularly with respect to reduced sodium intake, is arguably the most frequently recommended self‐care behavior and is endorsed by the national HF guidelines.^10^, ^11^ However, dietary recommendations are based largely on expert opinion and the limited randomized controlled trials that have been conducted to date have either focused on a single dietary component and/or have produced inconsistent findings.^12^, ^13^ Thus, there is an unmet clinical need to better understand the impact of lifestyle choices with respect to diet and nutrition in high‐risk HF patients who have been recently hospitalized.

Notably, there have been ongoing concerns that the novel coronavirus disease 2019 (COVID‐19) pandemic and the public health response (i.e., mitigation strategies) may have unintentionally limited access to community resources (e.g., fresh and nutritious food).^14^, ^15^, ^16^, ^17^, ^18^ Thus, to address this public health issue, we conducted a remotely administered survey using previously derived and validated questionnaires to describe (1) food security, (2) dietary quality and composition, (3) nutritional understanding, and (4) attitudes towards research among high‐risk HF patients during the pandemic.

## 2 METHODS

### 2.1 Source population

Kaiser Permanente Northern California (KPNC) is a large integrated healthcare delivery system currently providing comprehensive outpatient, emergency department, and inpatient care to >4.5 million members in northern and central California. The KPNC membership is highly representative of the local surrounding and statewide population in terms of age, gender, race/ethnicity, and socioeconomic status (SES). Nearly all aspects of care are captured through an integrated electronic health record (EHR) system, with key variables extracted and standardized for research in the Kaiser Permanente Virtual Data Warehouse.^19^, ^20^

This study was approved by the KPNC Institutional Review Board and patient consent was obtained.

### 2.2 Study eligibility

We initially identified all adult (≥18 years) KPNC members with a known diagnosis of HF on April 27, 2020 and at least one hospitalization for HF within the past year based on EHR data. The discharge diagnosis codes for HF have been validated in multiple healthcare delivery systems, with a positive predictive value ranging from ≥85% to 95%.^21^, ^22^ We excluded patients with <12 months of continuous prior health plan membership, a prior heart transplant or left ventricular assist device, admission to a skilled nursing facility within 30 days or hospice in the past 180 days, prior kidney replacement therapy, or a mailing address outside the KPNC geographic coverage area.

### 2.3 Survey design and administration

Our survey was comprised of four previously validated questionnaires covering the domains of food insecurity, diet quality and dietary habits, and nutrition knowledge, and additional items to ascertain basic demographic characteristics and attitudes towards research. The survey specifically assessed the following content areas:

- *Food Access and Security*—Researchers at the Children's HealthWatch team developed the Two‐Item Short Form of the Food Security Survey Module,^23^ which was first implemented in 1995 by The U.S. Department of Agriculture. Researchers have systematically evaluated the sensitivity, specificity, and bias of the Two‐Item Short Form of the Food Security Module relative to the longer 18‐item scale, and it provides an acceptable substitute with the added advantages that food‐insecure households can be more efficiently screened. Responses of “often true” or “sometimes true” are coded as affirmative (yes) and an affirmative response to either question is considered to be sufficient evidence of food insecurity. Importantly, the Two‐Item Short Form has been used successfully in mail‐out, take‐home, and on‐site self‐administered surveys and has served as the basis for hundreds of previously published studies.
- *Dietary Quality and Composition*—Understanding dietary diversity and quality is essential to assessing the nutritional needs of the general population and patients with known cardiovascular disease. However, most currently available dietary assessment tools are time‐consuming, expensive, and labor‐intensive, and/or limited by short‐term recall. The Rapid Eating Assessment for Participants—Shortened Version (REAP‐S)^24^ was incorporated as a straightforward, time‐efficient, and cost‐effective method to collect dietary information. The questionnaire consists of 13 items, which focus on eating habits, willingness to change, and individual food groups including fiber‐rich foods, fruits, vegetables, dairy products, processed meats, and high‐fat and high‐sugar foods. The REAP‐S inquires about food and drinks that respondents might have over an *average* week and asks them to rate the frequency as “usually/often,” “sometimes,” “rarely/never,” or “does not apply to me” for each item. Responses of “usually/often” receive 1 point, “sometimes” receive 2 points, and “rarely/never” or “does not apply to me” receive 3 points. Possible scores range from 13 to 39 with a higher score indicating a higher diet quality.
- *Nutritional Understanding*—Nutrition‐related knowledge addresses an individual's understanding of nutrition surrounding a person's eating behaviors. Recently published meta‐analyses and systematic reviews suggest that this domain is significantly associated with dietary behavior and nutritional intake.^25^, ^26^ Nutrition‐related knowledge data was collected using the Nutrition Knowledge Questionnaire, which includes 13 items on general nutrition knowledge including recommendations on total caloric intake and sources and consumption of selected nutrients.^27^ All items on the survey are closed‐ended and dichotomous or multiple choice. A score of 1 is assigned for each correct response and the total score is a sum ranging from 0 to 13 points with higher scores indicating better nutrition‐related knowledge.
- *Attitudes Towards Research*—A series of four questions were modified from the National Patient‐Centered Clinical Research Network (PCORnet) and pilot tested to assess a respondent's level of interest and willingness to be approached, consented, and enrolled in research. The questions specifically addressed prior participation in research, future willingness to participate in different types of research, interest in serving as an advisor and/or patient advocate for research studies, and preferred contact method(s) regarding potential research opportunities. All items were closed‐ended (i.e., dichotomous or multiple choice) and were individually analyzed using descriptive statistics.

The questionnaire was written in English and included both an electronic version hosted on the REDCap platform and a hardcopy version. We provided respondents with a $10 gift card to reimburse them for their time.

### 2.4 Handling of survey data

We sent surveys to patients' physical mailing addresses through the U.S. Postal Service and electronically (i.e., via email addresses registered with our healthcare delivery system), starting on May 18, 2020 for a total of two sequential contacts. Standard quality control measures were implemented to ensure the respondent was the intended member for both completed online and print surveys. Print surveys were edited by a study team member and data were entered into a clone of the online questionnaire that was used by participants who chose to complete the survey online. Our final cohort included patients who responded to the survey between May 18 and September 30, 2020. The date each patient's survey response was received was assigned as their index date. We performed a final exclusion of participants who were not health plan members on their assigned index date and those who were identified as having died before receipt of survey response.

### 2.5 Baseline covariates and follow‐up data

We also obtained data on demographic characteristics, comorbidities, vital signs, laboratory results, left ventricular ejection fraction (LVEF), and pharmacy dispensing using International Classification of Diseases 9th/10th Edition (ICD‐9/10) and current procedural terminology codes and relevant EHR data based on validated algorithms.^21^, ^22^

Clinical events of interest were all‐cause and HF‐specific hospitalizations and all‐cause mortality occurring at 90 and 180 days after the index date. The cause of all hospitalizations was based on the primary discharge diagnosis. These codes have been validated in multiple healthcare delivery systems and have a positive predictive value that ranges from ≥85% to 95%.^21^, ^22^ In addition, at KPNC, there is an exclusive relationship between the health plan, members, and providers such that nonnetwork referrals are extremely uncommon overall (i.e., <1% of clinical encounters). As a result, prior studies have shown that event capture through the EHR (i.e., emergency room visits, unplanned hospitalizations, and death) is >95%.^22^, ^28^, ^29^ Vital status was determined from multiple sources including EHR data (for deaths occurring in health plan facilities and member proxy reporting) and state death certificate information.

### 2.6 Statistical approach

We compared survey responses and baseline characteristics across SES, including income and education level, using analysis of variance for continuous variables, and *χ* ^2^ tests for categorical variables. We conducted Cox proportional hazards models to assess the association between diet quality and knowledge survey instrument responses and each outcome of interest at 90 and 180 days of follow‐up, with adjustment for age, sex, race, income, LVEF, systolic blood pressure, heart rate, b‐type natriuretic peptide (BNP), blood urea nitrogen, comorbidity point score—version 2,^30^ prior medication use (angiotensin‐converting‐enzyme inhibitor/angiotensin receptor blocker/angiotensin receptor‐neprilysin inhibitor [ACEi/ARBs/ARNIs], mineralocorticoid receptor antagonists [MRAs], β‐blockers, and diuretics); and targeted comorbidities (AF/AFL, acute myocardial infarction, unstable angina, and coronary revascularization). Each combination of survey instrument, outcome, and time point was modeled separately. We used SAS statistical software, version 9.4 for all analyses, with a two‐sided *p* < .05 as the threshold for statistical significance.

## 3 RESULTS

### 3.1 Cohort assembly and survey response

**Figure 1:**
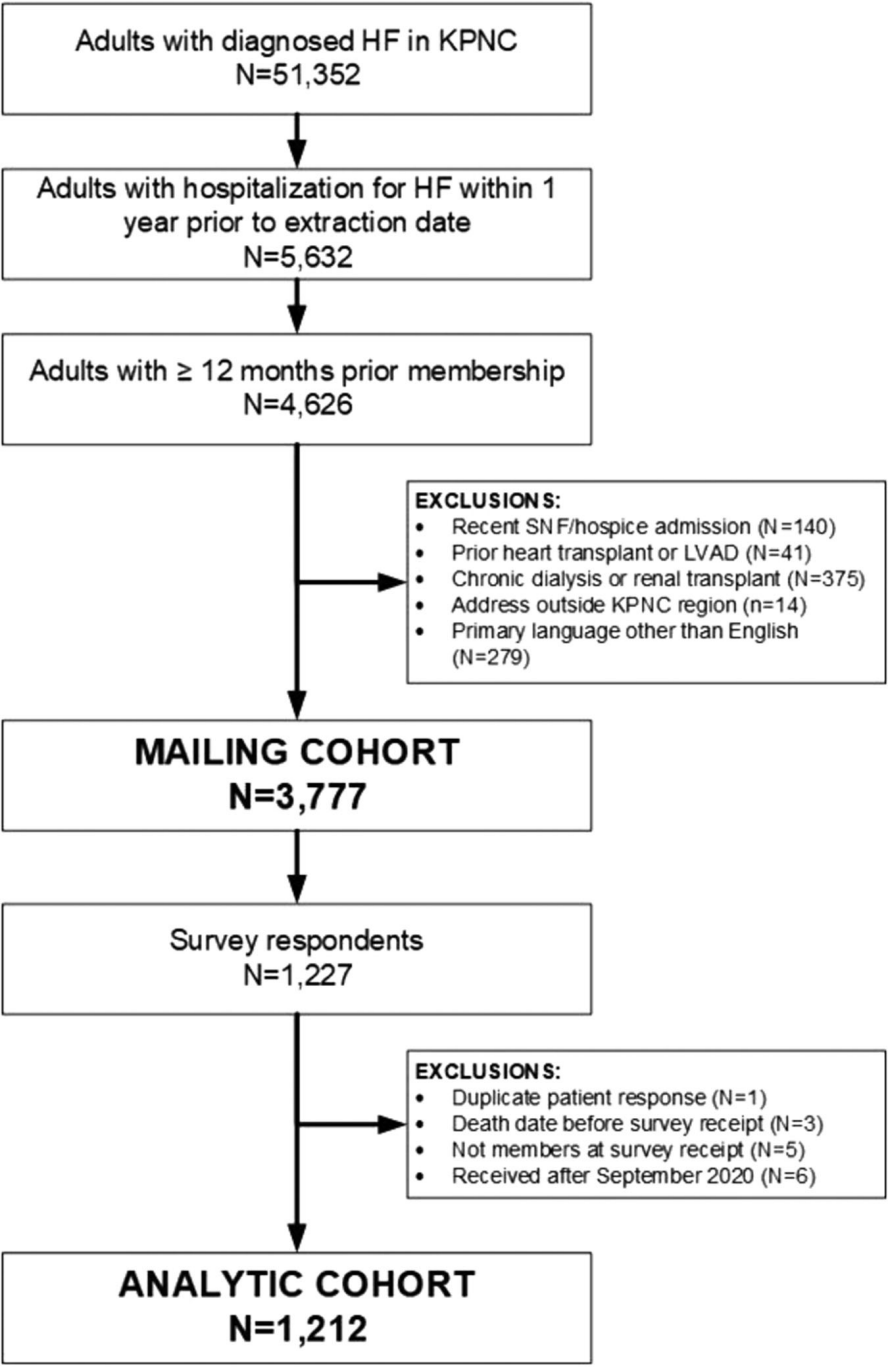
Consort diagram showing cohort assembly for survey eligibility and participant response following survey administration. HF heart failure; KPNC, Kaiser Permanente Northern California

We identified 51 352 adults with diagnosed HF as of April 27, 2020, with 5632 having been hospitalized for HF within the last year before their index date (Figure 1). After applying the remaining exclusion criteria, the eligible cohort included 3777 individuals. There was a total of 1212 survey respondents (32.5% of eligible cohort) between May 18, 2020 and September 30, 2020. Compared to nonrespondents, survey respondents were older and had a higher burden of selected comorbidities but were otherwise similar in terms of baseline sociodemographic and clinical characteristics (Table S1).

### 3.2 Baseline characteristics of survey respondents

The mean ± *SD* age of survey respondents was 77.9 ± 11.4% and 50.1% were women (Table S2). The median (interquartile range [IQR]) LVEF was 55% (40%–60%) and 42.2% of the cohort had a preserved LVEF. The median (IQR) BNP was 401 pg/ml (215–789 pg/ml). The burden of cardiac and noncardiac comorbidities was high, with 60.6% having AF/AFL, 52.7% had diabetes mellitus, and 50.7% with chronic kidney disease. Patients were well‐treated with β‐blockers, ACEis/ARBs/ARNis, and MRAs despite a low proportion of HF patients with a reduced LVEF. Differences in baseline clinical characteristics across SES (i.e., income and education level) are shown in Tables S3 and S4.

### 3.3 Food access and security

Based on the 2‐Item Short Form of the food security survey module, 15.1% of respondents reported being food secure, with 0.8% who did not respond (Table S2). The proportion of respondents reporting to be food insecure was higher for those with lower income (i.e., ≤$50 000 vs. >$50 000) and among participants for whom the highest level of education achieved was “less than high school” or “high school” compared to “some college” and “college graduate” (Tables S3 and S4).

### 3.4 Dietary quality and composition

**Figure 2:**
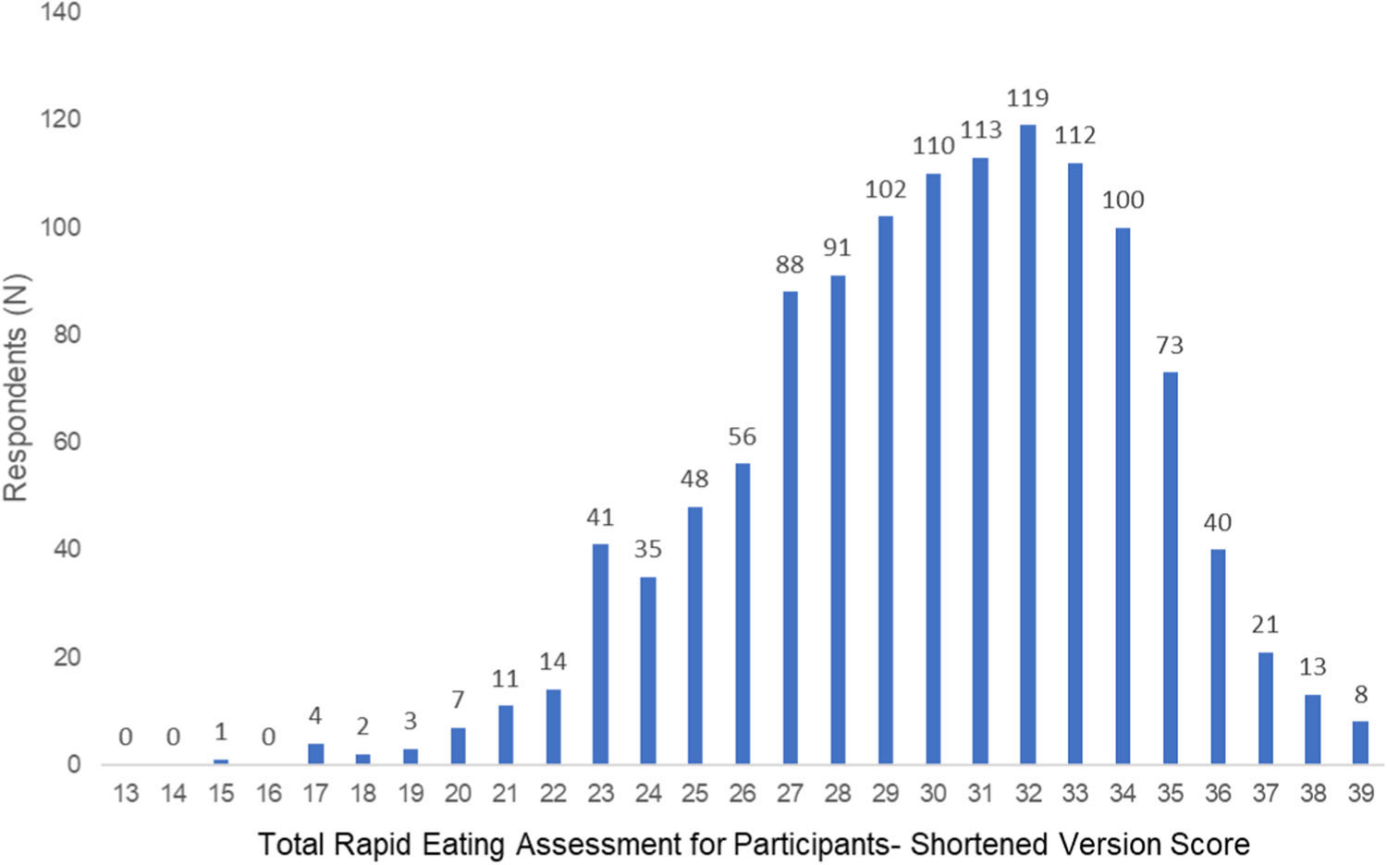
Distribution of scores among respondents for the rapid eating assessment for participants—a shortened version

The distribution of scores for the REAP‐S ranged from 13 to 39 (i.e., with higher scores indicating a higher diet quality) and are shown in Figure 2. There was an overall narrow distribution of scores based on SES (Tables S3 and S4).

### 3.5 Nutritional understanding

**Figure 3:**
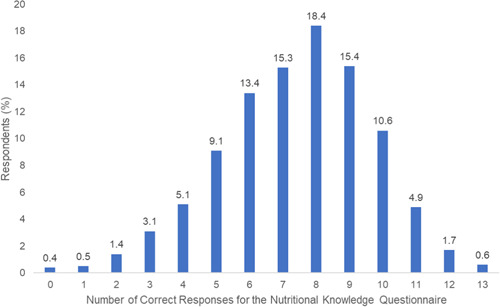
Distribution of number of questions answered correctly among respondents for the Nutritional Knowledge Questionnaire

The median (IQR) range of questions answered correctly was 8 (6–9) out of 13 questions for the overall cohort (Table S2 and Figure 3). In general, respondents who had an income >$50 000 and higher education level tended to answer more questions correctly (Tables S3 and S4).

### 3.6 Attitudes towards research

Overall, only 24.0% of survey respondents had previously participated in the research (Table S2). Although 65.0% of respondents were willing to consider participating in a future research survey, the proportion of respondents was willing to consider giving blood for research (32.2%), taking medication for research (14.4%), and changing behavior for research (27.1%) was substantially lower. In addition, only 7.6% and 25.5%, respectively, indicated they would either “Yes” or “Maybe” be interested in serving as a research advisor. Survey respondents indicated their preferred contact from most to least as the following: mailed letter (40.0%), e‐mail (22.0%), live phone call (13.3%), in‐person clinic visit (10.1%), text message (9.3%), and recorded phone call (2.8%). Overall, respondents with higher income and education levels expressed more willingness to participate and are contacted in the future regarding research opportunities (Tables S3 and S4).

### 3.7 Outcomes and interaction analyses

The incidence of HF hospitalizations, all‐cause hospitalizations, and death due to any cause at 90 and 180 days, respectively, were 4.1%/8.1%, 13.9%/25.2%, and 3.2%/7.9%. Only poor/fair versus excellent/very good/good (reference) self‐reported dietary health was associated with increased risk of all‐cause hospitalization at 90 and 180 days (Table 1). After multivariable adjustment, food security, dietary behaviors, and nutritional knowledge were not independently associated with SES.

**Table 1:** Adjusted associations between survey data and outcomes

	Death		Any hospitalization		HF hospitalization	
	HR (95% CI)	*p* Value	HR (95% CI)	*p* Value	HR (95% CI)	*p* Value
Outcomes at 90 days			
Food insecurity						
No food insecurity noted	(ref)		(ref)		(ref)	
Food insecurity noted	0.79 (0.24–2.58)	0.69	1.09 (0.71–1.69)	0.69	1.54 (0.78–3.05)	0.22
Nutritional knowledge						
Score at or over median	0.65 (0.29–1.45)	0.29	1.17 (0.85–1.63)	0.34	0.65 (0.33–1.28)	0.22
Score under median	(ref)		(ref)		(ref)	
Dietary behaviors (REAP)						
Score at or over median	0.88 (0.44–1.74)	0.70	1.23 (0.89–1.69)	0.20	1.04 (0.57–1.89)	0.91
Score under median	(ref)		(ref)		(ref)	
Outcomes at 180 days						
Food insecurity						
No food insecurity noted	(ref)		(ref)		(ref)	
Food insecurity noted	0.75 (0.38–1.49)	0.41	0.92 (0.65–1.30)	0.63	1.02 (0.59–1.74)	0.96
Nutritional knowledge						
Score at or over median	0.92 (0.58–1.47)	0.73	1.15 (0.90–1.47)	0.26	0.97 (0.62–1.52)	0.88
Score under median	(ref)		(ref)		(ref)	
Dietary behaviors (REAP)						
Score at or over median	0.84 (0.54–1.29)	0.42	0.98 (0.78–1.24)	0.87	1.00 (0.66–1.51)	0.99
Score under median	(ref)		(ref)		(ref)	

Abbreviations: CI, confidence interval; HF, heart failure; HR, hazard ratio.

## 4 DISCUSSION

To our knowledge, this is the first comprehensive survey addressing food access, diet quality and composition, nutritional understanding, and attitudes towards research in older adults with a high burden of cardiac and noncardiac comorbidities during the COVID‐19 pandemic. Notably, upwards of 15% of respondents screened positive for food insecurity, and only approximately 65% of respondents answered more than half of the items correctly on a questionnaire assessing nutritional understanding. In addition, although the majority of respondents indicated that would be willing to consider participating in future research, that proportion declined markedly for studies requiring blood draws, study drugs, and/or behavior change. In addition, lower income was associated with higher rates of food insecurity, and participants with a lower SES had a worse nutritional understanding. Finally, none of the functional domains assessed by this survey were independently associated with clinical outcomes in our cohort.

It may be unexpected that in an insured population approximately 15% of survey respondents screened positive for food insecurity. This is clinically relevant because there may be a misperception that well‐insured patients are less sensitive to modest fluctuations in out‐of‐pocket expenditures, but our data suggest that a sizable minority of patients may have difficulty affording basic necessities. We found that lower income and education levels had the strongest association with food insecurity. In addition, respondents who had a lower income and education level demonstrated a poorer understanding of basic nutritional concepts. In aggregate, these results suggest that even within a well‐insured population, there is room for improvement, and interventions directed at improving access to food, diet quality, and nutritional understanding may selectively target high‐risk populations, particularly those of lower SES.

These preliminary data on attitudes towards research also offer a glimpse into public perceptions at a time when lay individuals were engaging with the scientific process (i.e., COVID‐19 spread, emerging therapeutics, vaccine development, etc.) on an almost daily basis. Within this context, although less than a quarter of respondents had previously participated in a research study, nearly 65% indicated they would be willing to participate in another survey‐based study. However, there was much lower interest in participating in research involving giving blood (∼30%), taking a study medication (∼15%), or behavioral change (∼30%); however, these estimates may still be considered relatively high given the advanced age and high multimorbidity burden among survey respondents. It is noteworthy that the preferred initial mode of communication among participants was a mailed letter or email two‐to‐four‐fold versus a live phone call or in‐person clinic visit. This preference may be in part a reflection of the shelter‐in‐place orders that were in effect during most of the survey period but is likely still a generalizable finding given the magnitude of the difference. A final actionable insight is that respondents who had a higher income and education level were more likely to express a willingness to participate in future research opportunities. This is important as experimental protocols are part of the standard of care in many fields (i.e., oncology) and this may contribute to disparities in access, quality, and outcomes.

It is also worth noting that we did not observe an independent association between food access, diet quality, and nutritional knowledge, and all‐cause and HF‐related morbidity and mortality. However, there are several caveats to this observation. First, the point estimates of the hazard ratios were consistently in the direction of increased harm with food insecurity, poor dietary quality, and worse nutritional knowledge, and we may have been underpowered to detect statistically significant differences. Second, for some of the content areas covered by the survey such as food access, the proportion of abnormal values (i.e., food insecure) may have been too small and impeded our ability to find significant between‐group differences. Third, the proportion of survey respondents was unexpectedly lower given our organizations' extensive experience surveying members and the historically high response rates typically seen in an older demographic and patients with chronic medical conditions.^31^ As a result, this likely contributed as well to lower than expected event rates and reduced power in our study. Finally, it should be noted that food access is a relevant patient‐centered outcome and the association between diet and other lifestyle factors (i.e., exercise) has been strongly correlated with long‐term cardiovascular risk.^32^, ^33^

There are several limitations of the study. First, the study sample was recruited from a large integrated healthcare delivery system in northern California, and the results may not be generalizable to all other populations and practice settings. However, KPNC membership is diverse and highly representative of the local surrounding and statewide population in terms of age, gender, race/ethnicity, and SES. Second, the overall response rate was lower than anticipated introducing the possibility of selection bias. Despite this potential limitation, we were reassured that the baseline sociodemographic and clinical characteristics of the study sample were comparable to the source population. Third, some portions of the survey (i.e., attitudes towards research) have not been previously validated and should be considered hypothesis‐generating until they have been rigorously evaluated in different populations.

In conclusion, we identified potential barriers to food access, diet quality and composition, and nutritional understanding in a diverse and contemporary population of older adults with a high burden of medical comorbidities during the COVID‐19 pandemic. These findings were most prominent among individuals with lower SES. In addition, although most respondents indicated a willingness to consider participating in future research, the majority preferred observational rather than experimental (i.e., clinical trials) studies and mail and/or e‐mail as the primary mode of contact as opposed to phone or in‐person. Based on these insights, future efforts to improve access to healthy and nutritious food sources should leverage remote recruitment with a flexible and culturally sensitive intervention and selectively target at‐risk groups.

## CONFLICT OF INTERESTS

Andrew P. Ambrosy is supported by a Mentored Patient‐Oriented Research Career Development Award (K23HL150159) through the National Heart, Lung, and Blood Institute and has received relevant research support through grants to his institution from Amarin Pharma, Inc., Abbott, and Novartis. Alan S. Go has received relevant research support through grants to his institution from the National Heart, Lung and Blood Institute; National Institute of Diabetes, Digestive and Kidney Diseases; National Institute on Aging; Amarin Pharma, Inc.; Novartis; Janssen Research & Development; and CSL Behring. All other authors have declared no conflict of interests.

## AUTHOR CONTRIBUTIONS

**Andrew P. Ambrosy**: Conceptualization, methodology, investigation, writing (original draft), writing (reviewing and editing), and supervision. **Umar I. Malik**: Investigation, writing (original draft), writing (reviewing and editing). **Thomas K. Leong**: Investigation, formal analyses, and data curation. **Amanda R. Allen**: Data collection (development and management), writing (reviewing and editing). **Sue Hee Sung**: Project administration, supervision, investigation, and writing (reviewing and editing). **Alan S. Go**: Conceptualization, methodology, writing (original draft), writing (reviewing and editing), and supervision.

## Supporting information

Supplementary information.

## Data Availability

The data that support the findings of this study are available on request from the corresponding author. The data are not publicly available due to privacy or ethical restrictions.
