# Supplementary material for: Food security, diet quality, nutritional knowledge, and attitudes towards research in adults with heart failure during the COVID‐19 pandemic
Source: Clin Cardiol. 2022 Feb 2;45(2):180–8. doi: 10.1002/clc.23761 (PMC8860486; doi:10.1002/clc.23761)
Supplement: Supplementary file 1 — Supplementary information. [file CLC-45--s001.docx]

**Supplemental Table 1**. Baseline clinical characteristics for survey respondents vs. non-respondents.

|  | **Overall  (N=3,731)** | | **Respondents  (N=1,212)** | | **Non-respondents  (N=2,519)** | | **P** |
| --- | --- | --- | --- | --- | --- | --- | --- |
|  |  |  |  |  |  |  |  |
| **Demographic characteristics** |  |  |  |  |  |  |  |
| Mean (SD) age, yr | 75.5 | (13.4) | 77.9 | (11.4) | 74.4 | (14.2) | **<0.001** |
|  |  |  |  |  |  |  |  |
| **Left ventricular ejection fraction, %** |  |  |  |  |  |  |  |
| Median (IQR) | 54 | (35-60) | 55 | (40-60) | 52 | (35-60) | 0.06 |
|  |  |  |  |  |  |  | **<0.05** |
| Preserved, N (%) | 1,545 | (41.4) | 511 | (42.2) | 1,034 | (41.0) |  |
| Mid-range, N (%) | 410 | (11.0) | 135 | (11.1) | 275 | (10.9) |  |
| Reduced, N (%) | 739 | (19.8) | 206 | (17.0) | 533 | (21.2) |  |
| Missing, N (%) | 1,037 | (27.8) | 360 | (29.7) | 677 | (26.9) |  |
|  |  |  |  |  |  |  |  |
| **Medical history within 4 years** |  |  |  |  |  |  |  |
| Baseline COPS2 |  |  |  |  |  |  |  |
| Mean (SD) | 113 | (45) | 107 | (41) | 115 | (47) | **<0.001** |
| Median (IQR) | 109 | (81-138) | 105 | (79-132) | 111 | (82-144) | **<0.001** |
| Missing, N (%) | 0 | (0.0) | 0 | (0.0) | 0 | (0.0) |  |
|  |  |  |  |  |  |  |  |
| Baseline comorbidities, N (%) |  |  |  |  |  |  |  |
| Acute myocardial infarction | 406 | (10.9) | 142 | (11.7) | 264 | (10.5) | 0.26 |
| Unstable angina | 127 | (3.4) | 50 | (4.1) | 77 | (3.1) | 0.09 |
| Atrial fibrillation and/or atrial flutter | 2,063 | (55.3) | 735 | (60.6) | 1,328 | (52.7) | **<0.001** |
| Ventricular fibrillation or tachycardia | 138 | (3.7) | 44 | (3.6) | 94 | (3.7) | 0.88 |
| Ischemic stroke or transient ischemic attack | 241 | (6.5) | 67 | (5.5) | 174 | (6.9) | 0.11 |
| Peripheral artery disease | 383 | (10.3) | 124 | (10.2) | 259 | (10.3) | 0.96 |
| Mitral and/or aortic valvular disease | 981 | (26.3) | 348 | (28.7) | 633 | (25.1) | **<0.05** |
| Coronary artery bypass graft | 136 | (3.6) | 51 | (4.2) | 85 | (3.4) | 0.20 |
| Percutaneous coronary intervention | 320 | (8.6) | 125 | (10.3) | 195 | (7.7) | **<0.01** |
| Implantable cardioverter defibrillator | 201 | (5.4) | 73 | (6.0) | 128 | (5.1) | 0.23 |
| Pacemaker | 376 | (10.1) | 138 | (11.4) | 238 | (9.4) | 0.07 |
| Hypertension | 3,389 | (90.8) | 1,109 | (91.5) | 2,280 | (90.5) | 0.33 |
| Dyslipidemia | 3,326 | (89.1) | 1,107 | (91.3) | 2,219 | (88.1) | **<0.01** |
| Diabetes mellitus | 2,013 | (54.0) | 639 | (52.7) | 1,374 | (54.5) | 0.30 |
| Hospitalized bleeding | 371 | (9.9) | 124 | (10.2) | 247 | (9.8) | 0.68 |
| Chronic liver disease | 360 | (9.6) | 93 | (7.7) | 267 | (10.6) | **<0.01** |
| Chronic lung disease | 1,958 | (52.5) | 670 | (55.3) | 1,288 | (51.1) | **<0.05** |
| Hyperthyroidism | 119 | (3.2) | 38 | (3.1) | 81 | (3.2) | 0.90 |
| Hypothyroidism | 816 | (21.9) | 288 | (23.8) | 528 | (21.0) | 0.05 |
| Diagnosed dementia | 290 | (7.8) | 60 | (5.0) | 230 | (9.1) | **<0.001** |
| Diagnosed depression | 864 | (23.2) | 286 | (23.6) | 578 | (22.9) | 0.66 |
|  |  |  |  |  |  |  |  |
| **Medication use within 120 days, N (%)** |  |  |  |  |  |  |  |
| ACE inhibitor | 1,197 | (32.1) | 369 | (30.4) | 828 | (32.9) | 0.14 |
| Angiotensin II receptor blocker | 906 | (24.3) | 314 | (25.9) | 592 | (23.5) | 0.11 |
| Angiotensin receptor–neprilysin inhibitor | 101 | (2.7) | 36 | (3.0) | 65 | (2.6) | 0.49 |
| Beta blocker | 2,920 | (78.3) | 958 | (79.0) | 1,962 | (77.9) | 0.42 |
| Calcium channel blocker | 1,082 | (29.0) | 351 | (29.0) | 731 | (29.0) | 0.97 |
| Diuretic | 3,316 | (88.9) | 1,083 | (89.4) | 2,233 | (88.6) | 0.52 |
| Aldosterone receptor antagonist | 710 | (19.0) | 246 | (20.3) | 464 | (18.4) | 0.17 |
| Alpha blocker | 227 | (6.1) | 79 | (6.5) | 148 | (5.9) | 0.44 |
| Central alpha-adrenergic receptor agonists | 100 | (2.7) | 38 | (3.1) | 62 | (2.5) | 0.23 |
| Nitrates | 882 | (23.6) | 266 | (21.9) | 616 | (24.5) | 0.09 |
| Statin | 2,732 | (73.2) | 939 | (77.5) | 1,793 | (71.2) | **<0.001** |
| Other lipid-lowering agent | 72 | (1.9) | 38 | (3.1) | 34 | (1.3) | **<0.001** |
| Non-aspirin antiplatelet agent | 418 | (11.2) | 146 | (12.0) | 272 | (10.8) | 0.26 |
| Anticoagulant | 1,650 | (44.2) | 607 | (50.1) | 1,043 | (41.4) | **<0.001** |
| Diabetic therapy | 1,328 | (35.6) | 445 | (36.7) | 883 | (35.1) | 0.32 |
|  |  |  |  |  |  |  |  |
| **Vital signs within 4 years** |  |  |  |  |  |  |  |
| Tobacco use, N (%) |  |  |  |  |  |  | **<0.001** |
| None | 1,577 | (42.3) | 518 | (42.7) | 1,059 | (42.0) |  |
| Passive smoker | 19 | (0.5) | 8 | (0.7) | 11 | (0.4) |  |
| Former smoker | 1,954 | (52.4) | 655 | (54.0) | 1,299 | (51.6) |  |
| Current smoker | 181 | (4.9) | 31 | (2.6) | 150 | (6.0) |  |
| Systolic blood pressure, mmHg |  |  |  |  |  |  |  |
| Mean (SD) | 123.4 | (18.8) | 123.0 | (18.7) | 123.7 | (18.9) | 0.30 |
| Median (IQR) | 123.0 | (110.0-135.0) | 122.0 | (110.0-135.0) | 123.0 | (111.0-135.0) | 0.20 |
| Missing, N (%) | 143 | (3.8) | 23 | (1.9) | 120 | (4.8) |  |
| Diastolic blood pressure, mmHg |  |  |  |  |  |  |  |
| Mean (SD) | 64.7 | (12.5) | 63.3 | (12.4) | 65.4 | (12.5) | **<0.001** |
| Median (IQR) | 64.0 | (56.0-73.0) | 63.0 | (54.0-72.0) | 65.0 | (57.0-74.0) | **<0.001** |
| Missing, N (%) | 143 | (3.8) | 23 | (1.9) | 120 | (4.8) |  |
| Body mass index, kg/m2 |  |  |  |  |  |  |  |
| Mean (SD) | 30.6 | (9.1) | 30.0 | (8.5) | 30.9 | (9.4) | **<0.01** |
| Median (IQR) | 28.7 | (24.2-34.9) | 28.2 | (24.0-34.1) | 28.9 | (24.3-35.2) | **<0.05** |
| Missing, N (%) | 190 | (5.1) | 37 | (3.1) | 153 | (6.1) |  |
| Heart rate, b/min |  |  |  |  |  |  |  |
| Mean (SD) | 76.1 | (15.6) | 74.5 | (15.3) | 76.8 | (15.7) | **<0.001** |
| Median (IQR) | 74.0 | (65.0-86.0) | 72.0 | (64.0-84.0) | 75.0 | (66.0-87.0) | **<0.001** |
| Missing, N (%) | 144 | (3.9) | 24 | (2.0) | 120 | (4.8) |  |
|  |  |  |  |  |  |  |  |
| **Laboratory results within 1 year** |  |  |  |  |  |  |  |
| Estimated glomerular filtration rate, ml/min/1.73 m2 |  |  |  |  |  |  |  |
| Mean (SD) | 58.8 | (23.5) | 58.6 | (22.0) | 58.9 | (24.2) | 0.74 |
| Median (IQR) | 57.9 | (40.9-77.1) | 57.6 | (42.0-76.0) | 58.0 | (40.1-77.6) | 0.95 |
| Missing, N (%) | 278 | (7.5) | 52 | (4.3) | 226 | (9.0) |  |
| Blood urea nitrogen, mg/dL |  |  |  |  |  |  |  |
| Mean (SD) | 31.9 | (18.0) | 31.5 | (17.4) | 32.2 | (18.2) | 0.30 |
| Median (IQR) | 27.0 | (20.0-39.0) | 26.5 | (20.0-38.0) | 27.0 | (20.0-40.0) | 0.48 |
| Missing, N (%) | 822 | (22.0) | 236 | (19.5) | 586 | (23.3) |  |
| Brain natiuretic peptide, pg/mL |  |  |  |  |  |  |  |
| Mean (SD) | 627.2 | (755.3) | 571.5 | (689.9) | 656.3 | (786.0) | **<0.05** |
| Median (IQR) | 379.5 | (189.0-737.5) | 357.0 | (191.0-694.0) | 395.5 | (186.0-769.0) | 0.10 |
| Missing, N (%) | 1,963 | (52.6) | 606 | (50.0) | 1,357 | (53.9) |  |
| Brain natiuretic peptide, pg/mL, all settings |  |  |  |  |  |  |  |
| Mean (SD) | 734.5 | (848.9) | 629.6 | (694.1) | 783.3 | (908.1) | **<0.001** |
| Median (IQR) | 435.0 | (218.0-883.0) | 401.0 | (215.0-789.0) | 458.0 | (219.0-946.0) | **<0.001** |
| Missing, N (%) | 210 | (5.6) | 93 | (7.7) | 117 | (4.6) |  |

**Supplemental Table 2.** Baseline clinical characteristics and survey data for respondents.

|  | **All Survey Respondents**  **(N=1,212)** | |
| --- | --- | --- |
| **Demographic characteristics** |  |  |
| Mean (SD) age, yr | 77.9 | (11.4) |
| Self-reported gender, N (%) |  |  |
| Men | 603 | (49.8) |
| Women | 607 | (50.1) |
| Transgender men | 2 | (0.2) |
| Transgender women | 0 | (0.0) |
| Self-reported race/ethnicity, N (%) |  |  |
| White | 888 | (73.3) |
| Black/African American | 100 | (8.3) |
| Hispanic | 85 | (7.0) |
| Asian/Pacific Islander | 127 | (10.5) |
| Other/Unknown | 12 | (1.0) |
| Self-reported education, N (%) |  |  |
| Less than high school | 83 | (6.8) |
| High school or GED | 328 | (27.1) |
| Some college | 415 | (34.2) |
| Undergraduate degree or more | 356 | (29.4) |
| Missing or decline to state | 30 | (2.5) |
| Self-reported household income, N (%) |  |  |
| <$15,000 | 85 | (7.0) |
| $15,000-$25,000 | 112 | (9.2) |
| $25,000-$35,000 | 126 | (10.4) |
| $35,000-$50,000 | 158 | (13.0) |
| $50,000-$65,000 | 92 | (7.6) |
| $65,000-$80,000 | 105 | (8.7) |
| $80,000-$100,000 | 117 | (9.7) |
| $100,000-$150,000 | 90 | (7.4) |
| >$150,000 | 59 | (4.9) |
| Missing or decline to state | 268 | (22.1) |
| **Left ventricular ejection fraction, %** |  |  |
| Median (IQR) | 55 | (40-60) |
|  |  |  |
| Preserved, N (%) | 511 | (42.2) |
| Mid-range, N (%) | 135 | (11.1) |
| Reduced, N (%) | 206 | (17.0) |
| Missing, N (%) | 360 | (29.7) |
|  |  |  |
| **Medical history within 4 years** |  |  |
| Baseline COPS2 |  |  |
| Mean (SD) | 107 | (41) |
| Median (IQR) | 105 | (79-132) |
| Missing, N (%) | 0 | (0.0) |
|  |  |  |
| Baseline comorbidities, N (%) |  |  |
| Acute myocardial infarction | 142 | (11.7) |
| Unstable angina | 50 | (4.1) |
| Atrial fibrillation and/or atrial flutter | 735 | (60.6) |
| Ventricular fibrillation or tachycardia | 44 | (3.6) |
| Ischemic stroke or transient ischemic attack | 67 | (5.5) |
| Peripheral artery disease | 124 | (10.2) |
| Mitral and/or aortic valvular disease | 348 | (28.7) |
| Coronary artery bypass graft | 51 | (4.2) |
| Percutaneous coronary intervention | 125 | (10.3) |
| Implantable cardioverter defibrillator | 73 | (6.0) |
| Pacemaker | 138 | (11.4) |
| Hypertension | 1,109 | (91.5) |
| Dyslipidemia | 1,107 | (91.3) |
| Diabetes mellitus | 639 | (52.7) |
| Hospitalized bleeding | 124 | (10.2) |
| Chronic liver disease | 93 | (7.7) |
| Chronic lung disease | 670 | (55.3) |
| Hyperthyroidism | 38 | (3.1) |
| Hypothyroidism | 288 | (23.8) |
| Diagnosed dementia | 60 | (5.0) |
| Diagnosed depression | 286 | (23.6) |
|  |  |  |
| **Medication use within 120 days, N (%)** |  |  |
| ACE inhibitor | 369 | (30.4) |
| Angiotensin II receptor blocker | 314 | (25.9) |
| Angiotensin receptor–neprilysin inhibitor | 36 | (3.0) |
| Beta blocker | 958 | (79.0) |
| Calcium channel blocker | 351 | (29.0) |
| Diuretic | 1,083 | (89.4) |
| Aldosterone receptor antagonist | 246 | (20.3) |
| Alpha blocker | 79 | (6.5) |
| Central alpha-adrenergic receptor agonists | 38 | (3.1) |
| Nitrates | 266 | (21.9) |
| Statin | 939 | (77.5) |
| Other lipid-lowering agent | 38 | (3.1) |
| Non-aspirin antiplatelet agent | 146 | (12.0) |
| Anticoagulant | 607 | (50.1) |
| Diabetic therapy | 445 | (36.7) |
|  |  |  |
| **Vital signs within 4 years** |  |  |
| Tobacco use, N (%) |  |  |
| None | 518 | (42.7) |
| Passive smoker | 8 | (0.7) |
| Former smoker | 655 | (54.0) |
| Current smoker | 31 | (2.6) |
| Systolic blood pressure, mmHg |  |  |
| Mean (SD) | 123 | (18.7) |
| Median (IQR) | 122 | (110.0-135.0) |
| Missing, N (%) | 23 | (1.9) |
| Diastolic blood pressure, mmHg |  |  |
| Mean (SD) | 63.3 | (12.4) |
| Median (IQR) | 63 | (54.0-72.0) |
| Missing, N (%) | 23 | (1.9) |
| Body mass index, kg/m2 |  |  |
| Mean (SD) | 30 | (8.5) |
| Median (IQR) | 28.2 | (24.0-34.1) |
| Missing, N (%) | 37 | (3.1) |
| Heart rate, b/min |  |  |
| Mean (SD) | 74.5 | (15.3) |
| Median (IQR) | 72 | (64.0-84.0) |
| Missing, N (%) | 24 | (2.0) |
|  |  |  |
| **Laboratory results within 1 year** |  |  |
| Estimated glomerular filtration rate, ml/min/1.73 m2 |  |  |
| Mean (SD) | 58.6 | (22.0) |
| Median (IQR) | 57.6 | (42.0-76.0) |
| Missing, N (%) | 52 | (4.3) |
| Blood urea nitrogen, mg/dL |  |  |
| Mean (SD) | 31.5 | (17.4) |
| Median (IQR) | 26.5 | (20.0-38.0) |
| Missing, N (%) | 236 | (19.5) |
| Brain natriuretic peptide, pg/mL |  |  |
| Mean (SD) | 571.5 | (689.9) |
| Median (IQR) | 357 | (191.0-694.0) |
| Missing, N (%) | 606 | (50.0) |
| Brain natriuretic peptide, pg/mL, all settings |  |  |
| Mean (SD) | 629.6 | (694.1) |
| Median (IQR) | 401 | (215.0-789.0) |
| Missing, N (%) | 93 | (7.7) |
|  |  |  |
| **Diet and behavior responses** |  |  |
| Behavior - skipping breakfast |  |  |
| Often | 125 | (10.3) |
| Sometimes | 247 | (20.4) |
| Rarely | 826 | (68.2) |
| Missing | 14 | (1.2) |
| Behavior - 4 or more meals from restaurants per week |  |  |
| Often | 86 | (7.1) |
| Sometimes | 209 | (17.2) |
| Rarely | 875 | (72.2) |
| Missing | 42 | (3.5) |
| Behavior - less than 2 servings of high-fiber starch per day |  |  |
| Often | 319 | (26.3) |
| Sometimes | 507 | (41.8) |
| Rarely | 368 | (30.4) |
| Missing | 18 | (1.5) |
| Behavior - less than 2 servings of fruit per day |  |  |
| Often | 306 | (25.2) |
| Sometimes | 428 | (35.3) |
| Rarely | 464 | (38.3) |
| Missing | 14 | (1.2) |
| Behavior - less than 2 servings of vegetables per day |  |  |
| Often | 269 | (22.2) |
| Sometimes | 503 | (41.5) |
| Rarely | 419 | (34.6) |
| Missing | 21 | (1.7) |
| Behavior - less than 2 servings of dairy per day |  |  |
| Often | 383 | (31.6) |
| Sometimes | 418 | (34.5) |
| Rarely | 397 | (32.8) |
| Missing | 14 | (1.2) |
| Behavior - more than 8 ounces of meat per day |  |  |
| Often | 326 | (26.9) |
| Sometimes | 417 | (34.4) |
| Rarely | 461 | (38.0) |
| Missing | 8 | (0.7) |
| Behavior - use regular processed meats |  |  |
| Often | 95 | (7.8) |
| Sometimes | 396 | (32.7) |
| Rarely | 711 | (58.7) |
| Missing | 10 | (0.8) |
| Behavior - eat fried foods |  |  |
| Often | 77 | (6.4) |
| Sometimes | 472 | (38.9) |
| Rarely | 652 | (53.8) |
| Missing | 11 | (0.9) |
| Behavior - eat regular snack foods |  |  |
| Often | 109 | (9.0) |
| Sometimes | 420 | (34.7) |
| Rarely | 674 | (55.6) |
| Missing | 9 | (0.7) |
| Behavior - add butter, margarine or oil |  |  |
| Often | 388 | (32.0) |
| Sometimes | 464 | (38.3) |
| Rarely | 351 | (29.0) |
| Missing | 9 | (0.7) |
| Behavior - eat sweets |  |  |
| Often | 153 | (12.6) |
| Sometimes | 462 | (38.1) |
| Rarely | 584 | (48.2) |
| Missing | 13 | (1.1) |
| Behavior - drink 16 ounces of soda per day |  |  |
| Often | 112 | (9.2) |
| Sometimes | 206 | (17.0) |
| Rarely | 884 | (72.9) |
| Missing | 10 | (0.8) |
|  |  |  |
| Food insecurity index |  |  |
| Low risk | 1,019 | (84.1) |
| Worried | 66 | (5.4) |
| Ran out | 117 | (9.7) |
| Missing | 10 | (0.8) |
|  |  |  |
| **Knowledge responses** |  |  |
| Correct answers to knowledge questions, N (%) |  |  |
| There is more fiber in breads, rice, and vegetables than there is in meat, poultry, and eggs | 933 | (77.0) |
| Meat, fish, chicken, eggs, milk, legumes, grains, and vegetables contain protein | 1,116 | (92.1) |
| Prepackaged processed foods are a major source of salt in the Mediterranean diet | 733 | (60.5) |
| An orange contains more fiber than orange juice | 910 | (75.1) |
| Sugar contains many vitamins and minerals | 972 | (80.2) |
| The largest source of fat in the diet comes from animal foods | 771 | (63.6) |
| Milk, vegetables, grains, and fruits contain carbohydrates | 764 | (63.0) |
| To lower cholesterol in your blood, you only need to avoid foods that are high in cholesterol | 698 | (57.6) |
| Cooking at high temperatures diminishes the vitamins in foods | 729 | (60.1) |
| If a food is labeled “cholesterol free”, it must also be low in saturated fat | 581 | (47.9) |
| You can reduce the amount of fat in a recipe by substituting olive oil or corn oil for butter, lard, or chicken fat | 238 | (19.6) |
| One teaspoon of olive oil has about the same amount of calories as 1 teaspoon of butter | 318 | (26.2) |
| Cholesterol is found only in animal products | 211 | (17.4) |
|  |  |  |
| Number of correct answers |  |  |
| 0 answers | 5 | (0.4) |
| 1 answer | 6 | (0.5) |
| 2 answers | 17 | (1.4) |
| 3 answers | 38 | (3.1) |
| 4 answers | 62 | (5.1) |
| 5 answers | 110 | (9.1) |
| 6 answers | 163 | (13.4) |
| 7 answers | 185 | (15.3) |
| 8 answers | 223 | (18.4) |
| 9 answers | 187 | (15.4) |
| 10 answers | 129 | (10.6) |
| 11 answers | 59 | (4.9) |
| 12 answers | 21 | (1.7) |
| 13 answers | 7 | (0.6) |
|  |  |  |
| **Research responses** |  |  |
| Participated in prior research |  |  |
| No | 883 | (72.9) |
| Yes | 291 | (24.0) |
| Missing | 38 | (3.1) |
| Willing to consider future research survey |  |  |
| No | 210 | (17.3) |
| Yes | 788 | (65.0) |
| Missing | 214 | (17.7) |
| Willing to consider giving blood for research |  |  |
| No | 608 | (50.2) |
| Yes | 390 | (32.2) |
| Missing | 214 | (17.7) |
| Willing to consider taking medication for research |  |  |
| No | 824 | (68.0) |
| Yes | 174 | (14.4) |
| Missing | 214 | (17.7) |
| Willing to consider changing behavior for research |  |  |
| No | 669 | (55.2) |
| Yes | 329 | (27.1) |
| Missing | 214 | (17.7) |
| Interest in serving as a research advisor |  |  |
| No | 681 | (56.2) |
| Maybe | 309 | (25.5) |
| Yes | 92 | (7.6) |
| Checked all | 55 | (4.5) |
| Missing | 75 | (6.2) |
| Willing to be contacted by email |  |  |
| No | 650 | (53.6) |
| Yes | 267 | (22.0) |
| Missing | 295 | (24.3) |
| Willing to be contacted by mailed letter |  |  |
| No | 432 | (35.6) |
| Yes | 485 | (40.0) |
| Missing | 295 | (24.3) |
| Willing to be contacted by text message |  |  |
| No | 804 | (66.3) |
| Yes | 113 | (9.3) |
| Missing | 295 | (24.3) |
| Willing to be contacted by recorded phone call |  |  |
| No | 883 | (72.9) |
| Yes | 34 | (2.8) |
| Missing | 295 | (24.3) |
| Willing to be contacted by live phone call |  |  |
| No | 756 | (62.4) |
| Yes | 161 | (13.3) |
| Missing | 295 | (24.3) |
| Willing to be contacted by in-person clinic visit |  |  |
| No | 794 | (65.5) |
| Yes | 123 | (10.1) |
| Missing | 295 | (24.3) |
| Do not contact for future research |  |  |
| No | 924 | (76.2) |
| Yes | 288 | (23.8) |

**Supplemental Table 3**. Clinical characteristics and survey data stratified by income.

|  | **Income < $50,000** | **Income ≥ $50,000** | **Decline to state** | **P** |
| --- | --- | --- | --- | --- |
|  | **(N=481)** | **(N=463)** | **(N=268)** |  |
| **Demographic characteristics** |  |  |  |  |
| Self-reported gender, N (%) |  |  |  | **<0.001** |
| Men | 195 (40.5) | 285 (61.6) | 123 (45.9) |  |
| Women | 284 (59.0) | 178 (38.4) | 145 (54.1) |  |
| Transgender men | 2 (0.4) | 0 (0.0) | 0 (0.0) |  |
| Transgender women | 0 (0.0) | 0 (0.0) | 0 (0.0) |  |
| Self-reported race/ethnicity, N (%) |  |  |  | 0.61 |
| White | 346 (71.9) | 345 (74.5) | 197 (73.5) |  |
| Black/African American | 40 (8.3) | 36 (7.8) | 24 (9.0) |  |
| Hispanic | 31 (6.4) | 38 (8.2) | 16 (6.0) |  |
| Asian/Pacific Islander | 60 (12.5) | 40 (8.6) | 27 (10.1) |  |
| Other/Unknown | 4 (0.8) | 4 (0.9) | 4 (1.5) |  |
| Self-reported education, N (%) |  |  |  | **<0.001** |
| Less than high school | 54 (11.2) | 14 (3.0) | 15 (5.6) |  |
| High school or GED | 156 (32.4) | 87 (18.8) | 85 (31.7) |  |
| Some college | 181 (37.6) | 156 (33.7) | 78 (29.1) |  |
| Undergraduate degree or more | 84 (17.5) | 203 (43.8) | 69 (25.7) |  |
| Missing or decline to state | 6 (1.2) | 3 (0.6) | 21 (7.8) |  |
|  |  |  |  |  |
| **Diet and behavior responses** |  |  |  |  |
| Behavior - skipping breakfast |  |  |  | 0.08 |
| Often | 63 (13.1) | 38 (8.2) | 24 (9.0) |  |
| Sometimes | 106 (22.0) | 95 (20.5) | 46 (17.2) |  |
| Rarely | 308 (64.0) | 323 (69.8) | 195 (72.8) |  |
| Missing | 4 (0.8) | 7 (1.5) | 3 (1.1) |  |
| Behavior - 4 or more meals from restaurants per week |  |  |  | 0.21 |
| Often | 27 (5.6) | 44 (9.5) | 15 (5.6) |  |
| Sometimes | 83 (17.3) | 82 (17.7) | 44 (16.4) |  |
| Rarely | 351 (73.0) | 325 (70.2) | 199 (74.3) |  |
| Missing | 20 (4.2) | 12 (2.6) | 10 (3.7) |  |
| Behavior - less than 2 servings of high-fiber starch per day |  |  |  | **<0.05** |
| Often | 142 (29.5) | 108 (23.3) | 69 (25.7) |  |
| Sometimes | 199 (41.4) | 189 (40.8) | 119 (44.4) |  |
| Rarely | 128 (26.6) | 162 (35.0) | 78 (29.1) |  |
| Missing | 12 (2.5) | 4 (0.9) | 2 (0.7) |  |
| Behavior - less than 2 servings of fruit per day |  |  |  | 0.09 |
| Often | 137 (28.5) | 100 (21.6) | 69 (25.7) |  |
| Sometimes | 178 (37.0) | 160 (34.6) | 90 (33.6) |  |
| Rarely | 160 (33.3) | 198 (42.8) | 106 (39.6) |  |
| Missing | 6 (1.2) | 5 (1.1) | 3 (1.1) |  |
| Behavior - less than 2 servings of vegetables per day |  |  |  | **<0.05** |
| Often | 127 (26.4) | 87 (18.8) | 55 (20.5) |  |
| Sometimes | 204 (42.4) | 187 (40.4) | 112 (41.8) |  |
| Rarely | 141 (29.3) | 184 (39.7) | 94 (35.1) |  |
| Missing | 9 (1.9) | 5 (1.1) | 7 (2.6) |  |
| Behavior - less than 2 servings of dairy per day |  |  |  | 0.97 |
| Often | 158 (32.8) | 139 (30.0) | 86 (32.1) |  |
| Sometimes | 166 (34.5) | 162 (35.0) | 90 (33.6) |  |
| Rarely | 151 (31.4) | 157 (33.9) | 89 (33.2) |  |
| Missing | 6 (1.2) | 5 (1.1) | 3 (1.1) |  |
| Behavior - more than 8 ounces of meat per day |  |  |  | 0.82 |
| Often | 129 (26.8) | 125 (27.0) | 72 (26.9) |  |
| Sometimes | 172 (35.8) | 161 (34.8) | 84 (31.3) |  |
| Rarely | 177 (36.8) | 175 (37.8) | 109 (40.7) |  |
| Missing | 3 (0.6) | 2 (0.4) | 3 (1.1) |  |
| Behavior - use regular processed meats |  |  |  | 0.33 |
| Often | 40 (8.3) | 39 (8.4) | 16 (6.0) |  |
| Sometimes | 162 (33.7) | 151 (32.6) | 83 (31.0) |  |
| Rarely | 272 (56.5) | 271 (58.5) | 168 (62.7) |  |
| Missing | 7 (1.5) | 2 (0.4) | 1 (0.4) |  |
| Behavior - eat fried foods |  |  |  | 0.27 |
| Often | 33 (6.9) | 28 (6.0) | 16 (6.0) |  |
| Sometimes | 188 (39.1) | 192 (41.5) | 92 (34.3) |  |
| Rarely | 256 (53.2) | 241 (52.1) | 155 (57.8) |  |
| Missing | 4 (0.8) | 2 (0.4) | 5 (1.9) |  |
| Behavior - eat regular snack foods |  |  |  | 0.21 |
| Often | 35 (7.3) | 53 (11.4) | 21 (7.8) |  |
| Sometimes | 158 (32.8) | 168 (36.3) | 94 (35.1) |  |
| Rarely | 284 (59.0) | 239 (51.6) | 151 (56.3) |  |
| Missing | 4 (0.8) | 3 (0.6) | 2 (0.7) |  |
| Behavior - add butter, margarine or oil |  |  |  | 0.41 |
| Often | 148 (30.8) | 157 (33.9) | 83 (31.0) |  |
| Sometimes | 176 (36.6) | 173 (37.4) | 115 (42.9) |  |
| Rarely | 152 (31.6) | 130 (28.1) | 69 (25.7) |  |
| Missing | 5 (1.0) | 3 (0.6) | 1 (0.4) |  |
| Behavior - eat sweets |  |  |  | **<0.05** |
| Often | 75 (15.6) | 52 (11.2) | 26 (9.7) |  |
| Sometimes | 186 (38.7) | 181 (39.1) | 95 (35.4) |  |
| Rarely | 218 (45.3) | 225 (48.6) | 141 (52.6) |  |
| Missing | 2 (0.4) | 5 (1.1) | 6 (2.2) |  |
| Behavior - drink 16 ounces of soda per day |  |  |  | 0.06 |
| Often | 55 (11.4) | 37 (8.0) | 20 (7.5) |  |
| Sometimes | 96 (20.0) | 69 (14.9) | 41 (15.3) |  |
| Rarely | 325 (67.6) | 354 (76.5) | 205 (76.5) |  |
| Missing | 5 (1.0) | 3 (0.6) | 2 (0.7) |  |
|  |  |  |  |  |
| Food insecurity index |  |  |  | **<0.001** |
| Low risk | 355 (73.8) | 428 (92.4) | 236 (88.1) |  |
| Worried | 34 (7.1) | 20 (4.3) | 12 (4.5) |  |
| Ran out | 90 (18.7) | 11 (2.4) | 16 (6.0) |  |
| Missing | 2 (0.4) | 4 (0.9) | 4 (1.5) |  |
|  |  |  |  |  |
| **Knowledge responses** |  |  |  |  |
| Correct answers to knowledge questions, N (%) |  |  |  |  |
| There is more fiber in breads, rice, and vegetables than there is in meat, poultry, and eggs | 353 (73.4) | 379 (81.9) | 201 (75.0) | **<0.01** |
| Meat, fish, chicken, eggs, milk, legumes, grains, and vegetables contain protein | 442 (91.9) | 433 (93.5) | 241 (89.9) | 0.22 |
| Prepackaged processed foods are a major source of salt in the Mediterranean diet | 294 (61.1) | 279 (60.3) | 160 (59.7) | 0.92 |
| An orange contains more fiber than orange juice | 343 (71.3) | 371 (80.1) | 196 (73.1) | **<0.01** |
| Sugar contains many vitamins and minerals | 374 (77.8) | 389 (84.0) | 209 (78.0) | **<0.05** |
| The largest source of fat in the diet comes from animal foods | 284 (59.0) | 315 (68.0) | 172 (64.2) | **<0.05** |
| Milk, vegetables, grains, and fruits contain carbohydrates | 287 (59.7) | 319 (68.9) | 158 (59.0) | **<0.01** |
| To lower cholesterol in your blood, you only need to avoid foods that are high in cholesterol | 254 (52.8) | 303 (65.4) | 141 (52.6) | **<0.001** |
| Cooking at high temperatures diminishes the vitamins in foods | 275 (57.2) | 295 (63.7) | 159 (59.3) | 0.12 |
| If a food is labeled “cholesterol free”, it must also be low in saturated fat | 234 (48.6) | 224 (48.4) | 123 (45.9) | 0.75 |
| You can reduce the amount of fat in a recipe by substituting olive oil or corn oil for butter, lard, or chicken fat | 95 (19.8) | 94 (20.3) | 49 (18.3) | 0.80 |
| One teaspoon of olive oil has about the same amount of calories as 1 teaspoon of butter | 126 (26.2) | 126 (27.2) | 66 (24.6) | 0.75 |
| Cholesterol is found only in animal products | 77 (16.0) | 84 (18.1) | 50 (18.7) | 0.57 |
|  |  |  |  |  |
| Number of correct answers |  |  |  | **<0.05** |
| 0 answers | 3 (0.6) | 1 (0.2) | 1 (0.4) |  |
| 1 answer | 3 (0.6) | 2 (0.4) | 1 (0.4) |  |
| 2 answers | 9 (1.9) | 3 (0.6) | 5 (1.9) |  |
| 3 answers | 22 (4.6) | 5 (1.1) | 11 (4.1) |  |
| 4 answers | 29 (6.0) | 15 (3.2) | 18 (6.7) |  |
| 5 answers | 48 (10.0) | 34 (7.3) | 28 (10.4) |  |
| 6 answers | 67 (13.9) | 64 (13.8) | 32 (11.9) |  |
| 7 answers | 77 (16.0) | 70 (15.1) | 38 (14.2) |  |
| 8 answers | 81 (16.8) | 94 (20.3) | 48 (17.9) |  |
| 9 answers | 57 (11.9) | 76 (16.4) | 54 (20.1) |  |
| 10 answers | 55 (11.4) | 56 (12.1) | 18 (6.7) |  |
| 11 answers | 20 (4.2) | 31 (6.7) | 8 (3.0) |  |
| 12 answers | 7 (1.5) | 9 (1.9) | 5 (1.9) |  |
| 13 answers | 3 (0.6) | 3 (0.6) | 1 (0.4) |  |
|  |  |  |  |  |
| **Research responses** |  |  |  |  |
| Participated in prior research |  |  |  | **<0.05** |
| No | 368 (76.5) | 312 (67.4) | 203 (75.7) |  |
| Yes | 99 (20.6) | 136 (29.4) | 56 (20.9) |  |
| Missing | 14 (2.9) | 15 (3.2) | 9 (3.4) |  |
|  |  |  |  |  |
| Willing to consider future research survey |  |  |  | **<0.001** |
| No | 96 (20.0) | 73 (15.8) | 41 (15.3) |  |
| Yes | 306 (63.6) | 335 (72.4) | 147 (54.9) |  |
| Missing | 79 (16.4) | 55 (11.9) | 80 (29.9) |  |
| Willing to consider giving blood for research |  |  |  | **<0.001** |
| No | 266 (55.3) | 217 (46.9) | 125 (46.6) |  |
| Yes | 136 (28.3) | 191 (41.3) | 63 (23.5) |  |
| Missing | 79 (16.4) | 55 (11.9) | 80 (29.9) |  |
| Willing to consider taking medication for research |  |  |  | **<0.001** |
| No | 333 (69.2) | 325 (70.2) | 166 (61.9) |  |
| Yes | 69 (14.3) | 83 (17.9) | 22 (8.2) |  |
| Missing | 79 (16.4) | 55 (11.9) | 80 (29.9) |  |
| Willing to consider changing behavior for research |  |  |  | **<0.001** |
| No | 284 (59.0) | 265 (57.2) | 120 (44.8) |  |
| Yes | 118 (24.5) | 143 (30.9) | 68 (25.4) |  |
| Missing | 79 (16.4) | 55 (11.9) | 80 (29.9) |  |
| Interest in serving as a research advisor |  |  |  | **<0.001** |
| No | 291 (60.5) | 231 (49.9) | 159 (59.3) |  |
| Maybe | 117 (24.3) | 135 (29.2) | 57 (21.3) |  |
| Yes | 30 (6.2) | 47 (10.2) | 15 (5.6) |  |
| Checked all | 23 (4.8) | 23 (5.0) | 9 (3.4) |  |
| Missing | 20 (4.2) | 27 (5.8) | 28 (10.4) |  |
|  |  |  |  |  |
| Willing to be contacted by email |  |  |  | **<0.001** |
| No | 261 (54.3) | 239 (51.6) | 150 (56.0) |  |
| Yes | 90 (18.7) | 147 (31.7) | 30 (11.2) |  |
| Missing | 130 (27.0) | 77 (16.6) | 88 (32.8) |  |
| Willing to be contacted by mailed letter |  |  |  | **<0.001** |
| No | 148 (30.8) | 188 (40.6) | 96 (35.8) |  |
| Yes | 203 (42.2) | 198 (42.8) | 84 (31.3) |  |
| Missing | 130 (27.0) | 77 (16.6) | 88 (32.8) |  |
| Willing to be contacted by text message |  |  |  | **<0.001** |
| No | 297 (61.7) | 338 (73.0) | 169 (63.1) |  |
| Yes | 54 (11.2) | 48 (10.4) | 11 (4.1) |  |
| Missing | 130 (27.0) | 77 (16.6) | 88 (32.8) |  |
| Willing to be contacted by recorded phone call |  |  |  | **<0.001** |
| No | 339 (70.5) | 367 (79.3) | 177 (66.0) |  |
| Yes | 12 (2.5) | 19 (4.1) | 3 (1.1) |  |
| Missing | 130 (27.0) | 77 (16.6) | 88 (32.8) |  |
| Willing to be contacted by live phone call |  |  |  | **<0.001** |
| No | 291 (60.5) | 311 (67.2) | 154 (57.5) |  |
| Yes | 60 (12.5) | 75 (16.2) | 26 (9.7) |  |
| Missing | 130 (27.0) | 77 (16.6) | 88 (32.8) |  |
| Willing to be contacted by in-person clinic visit |  |  |  | **<0.001** |
| No | 298 (62.0) | 333 (71.9) | 163 (60.8) |  |
| Yes | 53 (11.0) | 53 (11.4) | 17 (6.3) |  |
| Missing | 130 (27.0) | 77 (16.6) | 88 (32.8) |  |
| Do not contact for future research |  |  |  | **<0.001** |
| No | 351 (73.0) | 386 (83.4) | 187 (69.8) |  |
| Yes | 130 (27.0) | 77 (16.6) | 81 (30.2) |  |

**Supplemental Table 4**. Clinical characteristics and survey data stratified by education.

|  | **Less than high school** | **High school** | **Some college** | **College graduate** | **Unknown** | **P** |
| --- | --- | --- | --- | --- | --- | --- |
|  | **(N=83)** | **(N=328)** | **(N=415)** | **(N=356)** | **(N=30)** |  |
| **Demographic characteristics** |  |  |  |  |  |  |
| Self-reported gender, N (%) |  |  |  |  |  | 0.31 |
| Men | 37 (44.6) | 156 (47.6) | 196 (47.2) | 198 (55.6) | 16 (53.3) |  |
| Women | 46 (55.4) | 171 (52.1) | 219 (52.8) | 157 (44.1) | 14 (46.7) |  |
| Transgender men | 0 (0.0) | 1 (0.3) | 0 (0.0) | 1 (0.3) | 0 (0.0) |  |
| Transgender women | 0 (0.0) | 0 (0.0) | 0 (0.0) | 0 (0.0) | 0 (0.0) |  |
| Self-reported race/ethnicity, N (%) |  |  |  |  |  | **<0.01** |
| White | 57 (68.7) | 245 (74.7) | 305 (73.5) | 260 (73.0) | 21 (70.0) |  |
| Black/African American | 5 (6.0) | 22 (6.7) | 46 (11.1) | 24 (6.7) | 3 (10.0) |  |
| Hispanic | 9 (10.8) | 31 (9.5) | 24 (5.8) | 21 (5.9) | 0 (0.0) |  |
| Asian/Pacific Islander | 9 (10.8) | 27 (8.2) | 35 (8.4) | 50 (14.0) | 6 (20.0) |  |
| Other/Unknown | 3 (3.6) | 3 (0.9) | 5 (1.2) | 1 (0.3) | 0 (0.0) |  |
| Self-reported household income, N (%) |  |  |  |  |  | **<0.001** |
| <$15,000 | 19 (22.9) | 18 (5.5) | 34 (8.2) | 12 (3.4) | 2 (6.7) |  |
| $15,000-$25,000 | 9 (10.8) | 45 (13.7) | 39 (9.4) | 18 (5.1) | 1 (3.3) |  |
| $25,000-$35,000 | 10 (12.0) | 44 (13.4) | 53 (12.8) | 18 (5.1) | 1 (3.3) |  |
| $35,000-$50,000 | 16 (19.3) | 49 (14.9) | 55 (13.3) | 36 (10.1) | 2 (6.7) |  |
| $50,000-$65,000 | 7 (8.4) | 25 (7.6) | 35 (8.4) | 24 (6.7) | 1 (3.3) |  |
| $65,000-$80,000 | 2 (2.4) | 25 (7.6) | 36 (8.7) | 40 (11.2) | 2 (6.7) |  |
| $80,000-$100,000 | 1 (1.2) | 20 (6.1) | 38 (9.2) | 58 (16.3) | 0 (0.0) |  |
| $100,000-$150,000 | 2 (2.4) | 15 (4.6) | 31 (7.5) | 42 (11.8) | 0 (0.0) |  |
| >$150,000 | 2 (2.4) | 2 (0.6) | 16 (3.9) | 39 (11.0) | 0 (0.0) |  |
| Missing or decline to state | 15 (18.1) | 85 (25.9) | 78 (18.8) | 69 (19.4) | 21 (70.0) |  |
|  |  |  |  |  |  |  |
| **Diet and behavior responses** |  |  |  |  |  |  |
| Behavior - skipping breakfast |  |  |  |  |  | 0.14 |
| Often | 11 (13.3) | 42 (12.8) | 39 (9.4) | 30 (8.4) | 3 (10.0) |  |
| Sometimes | 14 (16.9) | 66 (20.1) | 102 (24.6) | 60 (16.9) | 5 (16.7) |  |
| Rarely | 57 (68.7) | 217 (66.2) | 267 (64.3) | 264 (74.2) | 21 (70.0) |  |
| Missing | 1 (1.2) | 3 (0.9) | 7 (1.7) | 2 (0.6) | 1 (3.3) |  |
| Behavior - 4 or more meals from restaurants per week |  |  |  |  |  | 0.37 |
| Often | 6 (7.2) | 23 (7.0) | 32 (7.7) | 22 (6.2) | 3 (10.0) |  |
| Sometimes | 12 (14.5) | 63 (19.2) | 68 (16.4) | 64 (18.0) | 2 (6.7) |  |
| Rarely | 61 (73.5) | 233 (71.0) | 294 (70.8) | 264 (74.2) | 23 (76.7) |  |
| Missing | 4 (4.8) | 9 (2.7) | 21 (5.1) | 6 (1.7) | 2 (6.7) |  |
| Behavior - less than 2 servings of high-fiber starch per day |  |  |  |  |  | **<0.05** |
| Often | 28 (33.7) | 92 (28.0) | 102 (24.6) | 91 (25.6) | 6 (20.0) |  |
| Sometimes | 27 (32.5) | 151 (46.0) | 181 (43.6) | 130 (36.5) | 18 (60.0) |  |
| Rarely | 27 (32.5) | 79 (24.1) | 125 (30.1) | 132 (37.1) | 5 (16.7) |  |
| Missing | 1 (1.2) | 6 (1.8) | 7 (1.7) | 3 (0.8) | 1 (3.3) |  |
| Behavior - less than 2 servings of fruit per day |  |  |  |  |  | **<0.001** |
| Often | 32 (38.6) | 104 (31.7) | 97 (23.4) | 68 (19.1) | 5 (16.7) |  |
| Sometimes | 28 (33.7) | 108 (32.9) | 162 (39.0) | 117 (32.9) | 13 (43.3) |  |
| Rarely | 21 (25.3) | 109 (33.2) | 154 (37.1) | 168 (47.2) | 12 (40.0) |  |
| Missing | 2 (2.4) | 7 (2.1) | 2 (0.5) | 3 (0.8) | 0 (0.0) |  |
| Behavior - less than 2 servings of vegetables per day |  |  |  |  |  | **<0.001** |
| Often | 26 (31.3) | 90 (27.4) | 88 (21.2) | 61 (17.1) | 4 (13.3) |  |
| Sometimes | 35 (42.2) | 137 (41.8) | 182 (43.9) | 139 (39.0) | 10 (33.3) |  |
| Rarely | 18 (21.7) | 96 (29.3) | 140 (33.7) | 150 (42.1) | 15 (50.0) |  |
| Missing | 4 (4.8) | 5 (1.5) | 5 (1.2) | 6 (1.7) | 1 (3.3) |  |
| Behavior - less than 2 servings of dairy per day |  |  |  |  |  | 0.40 |
| Often | 34 (41.0) | 100 (30.5) | 119 (28.7) | 124 (34.8) | 6 (20.0) |  |
| Sometimes | 27 (32.5) | 117 (35.7) | 143 (34.5) | 119 (33.4) | 12 (40.0) |  |
| Rarely | 20 (24.1) | 108 (32.9) | 147 (35.4) | 110 (30.9) | 12 (40.0) |  |
| Missing | 2 (2.4) | 3 (0.9) | 6 (1.4) | 3 (0.8) | 0 (0.0) |  |
| Behavior - more than 8 ounces of meat per day |  |  |  |  |  | 0.94 |
| Often | 26 (31.3) | 88 (26.8) | 109 (26.3) | 94 (26.4) | 9 (30.0) |  |
| Sometimes | 28 (33.7) | 119 (36.3) | 148 (35.7) | 114 (32.0) | 8 (26.7) |  |
| Rarely | 29 (34.9) | 119 (36.3) | 154 (37.1) | 146 (41.0) | 13 (43.3) |  |
| Missing | 0 (0.0) | 2 (0.6) | 4 (1.0) | 2 (0.6) | 0 (0.0) |  |
| Behavior - use regular processed meats |  |  |  |  |  | 0.21 |
| Often | 9 (10.8) | 27 (8.2) | 38 (9.2) | 20 (5.6) | 1 (3.3) |  |
| Sometimes | 24 (28.9) | 121 (36.9) | 140 (33.7) | 103 (28.9) | 8 (26.7) |  |
| Rarely | 49 (59.0) | 179 (54.6) | 232 (55.9) | 230 (64.6) | 21 (70.0) |  |
| Missing | 1 (1.2) | 1 (0.3) | 5 (1.2) | 3 (0.8) | 0 (0.0) |  |
| Behavior - eat fried foods |  |  |  |  |  | **<0.05** |
| Often | 5 (6.0) | 17 (5.2) | 36 (8.7) | 17 (4.8) | 2 (6.7) |  |
| Sometimes | 36 (43.4) | 140 (42.7) | 171 (41.2) | 117 (32.9) | 8 (26.7) |  |
| Rarely | 42 (50.6) | 168 (51.2) | 203 (48.9) | 220 (61.8) | 19 (63.3) |  |
| Missing | 0 (0.0) | 3 (0.9) | 5 (1.2) | 2 (0.6) | 1 (3.3) |  |
| Behavior - eat regular snack foods |  |  |  |  |  | 0.12 |
| Often | 4 (4.8) | 34 (10.4) | 44 (10.6) | 23 (6.5) | 4 (13.3) |  |
| Sometimes | 34 (41.0) | 120 (36.6) | 150 (36.1) | 109 (30.6) | 7 (23.3) |  |
| Rarely | 45 (54.2) | 173 (52.7) | 217 (52.3) | 220 (61.8) | 19 (63.3) |  |
| Missing | 0 (0.0) | 1 (0.3) | 4 (1.0) | 4 (1.1) | 0 (0.0) |  |
| Behavior - add butter, margarine or oil |  |  |  |  |  | 0.16 |
| Often | 24 (28.9) | 121 (36.9) | 132 (31.8) | 107 (30.1) | 4 (13.3) |  |
| Sometimes | 36 (43.4) | 118 (36.0) | 169 (40.7) | 129 (36.2) | 12 (40.0) |  |
| Rarely | 23 (27.7) | 86 (26.2) | 112 (27.0) | 116 (32.6) | 14 (46.7) |  |
| Missing | 0 (0.0) | 3 (0.9) | 2 (0.5) | 4 (1.1) | 0 (0.0) |  |
| Behavior - eat sweets |  |  |  |  |  | **<0.05** |
| Often | 12 (14.5) | 52 (15.9) | 53 (12.8) | 31 (8.7) | 5 (16.7) |  |
| Sometimes | 41 (49.4) | 131 (39.9) | 153 (36.9) | 128 (36.0) | 9 (30.0) |  |
| Rarely | 29 (34.9) | 144 (43.9) | 202 (48.7) | 193 (54.2) | 16 (53.3) |  |
| Missing | 1 (1.2) | 1 (0.3) | 7 (1.7) | 4 (1.1) | 0 (0.0) |  |
| Behavior - drink 16 ounces of soda per day |  |  |  |  |  | 0.15 |
| Often | 11 (13.3) | 40 (12.2) | 36 (8.7) | 23 (6.5) | 2 (6.7) |  |
| Sometimes | 12 (14.5) | 58 (17.7) | 83 (20.0) | 49 (13.8) | 4 (13.3) |  |
| Rarely | 60 (72.3) | 227 (69.2) | 292 (70.4) | 281 (78.9) | 24 (80.0) |  |
| Missing | 0 (0.0) | 3 (0.9) | 4 (1.0) | 3 (0.8) | 0 (0.0) |  |
|  |  |  |  |  |  |  |
| Food insecurity index |  |  |  |  |  | **<0.001** |
| Low risk | 61 (73.5) | 263 (80.2) | 341 (82.2) | 329 (92.4) | 25 (83.3) |  |
| Worried | 6 (7.2) | 20 (6.1) | 26 (6.3) | 12 (3.4) | 2 (6.7) |  |
| Ran out | 16 (19.3) | 40 (12.2) | 48 (11.6) | 10 (2.8) | 3 (10.0) |  |
| Missing | 0 (0.0) | 5 (1.5) | 0 (0.0) | 5 (1.4) | 0 (0.0) |  |
|  |  |  |  |  |  |  |
| **Knowledge responses** |  |  |  |  |  |  |
| Correct answers to knowledge questions, N (%) |  |  |  |  |  |  |
| There is more fiber in breads, rice, and vegetables than there is in meat, poultry, and eggs | 49 (59.0) | 241 (73.5) | 320 (77.1) | 300 (84.3) | 23 (76.7) | **<0.001** |
| Meat, fish, chicken, eggs, milk, legumes, grains, and vegetables contain protein | 72 (86.7) | 291 (88.7) | 394 (94.9) | 333 (93.5) | 26 (86.7) | **<0.01** |
| Prepackaged processed foods are a major source of salt in the Mediterranean diet | 46 (55.4) | 200 (61.0) | 258 (62.2) | 213 (59.8) | 16 (53.3) | 0.71 |
| An orange contains more fiber than orange juice | 54 (65.1) | 223 (68.0) | 314 (75.7) | 295 (82.9) | 24 (80.0) | **<0.001** |
| Sugar contains many vitamins and minerals | 65 (78.3) | 243 (74.1) | 330 (79.5) | 314 (88.2) | 20 (66.7) | **<0.001** |
| The largest source of fat in the diet comes from animal foods | 42 (50.6) | 197 (60.1) | 253 (61.0) | 259 (72.8) | 20 (66.7) | **<0.001** |
| Milk, vegetables, grains, and fruits contain carbohydrates | 42 (50.6) | 187 (57.0) | 259 (62.4) | 259 (72.8) | 17 (56.7) | **<0.001** |
| To lower cholesterol in your blood, you only need to avoid foods that are high in cholesterol | 32 (38.6) | 151 (46.0) | 259 (62.4) | 241 (67.7) | 15 (50.0) | **<0.001** |
| Cooking at high temperatures diminishes the vitamins in foods | 45 (54.2) | 186 (56.7) | 237 (57.1) | 248 (69.7) | 13 (43.3) | **<0.001** |
| If a food is labeled “cholesterol free”, it must also be low in saturated fat | 29 (34.9) | 155 (47.3) | 200 (48.2) | 187 (52.5) | 10 (33.3) | **<0.05** |
| You can reduce the amount of fat in a recipe by substituting olive oil or corn oil for butter, lard, or chicken fat | 15 (18.1) | 71 (21.6) | 80 (19.3) | 65 (18.3) | 7 (23.3) | 0.79 |
| One teaspoon of olive oil has about the same amount of calories as 1 teaspoon of butter | 20 (24.1) | 76 (23.2) | 110 (26.5) | 106 (29.8) | 6 (20.0) | 0.32 |
| Cholesterol is found only in animal products | 11 (13.3) | 50 (15.2) | 64 (15.4) | 81 (22.8) | 5 (16.7) | **<0.05** |
|  |  |  |  |  |  |  |
| Number of correct answers |  |  |  |  |  | **<0.001** |
| 0 answers | 2 (2.4) | 1 (0.3) | 1 (0.2) | 1 (0.3) | 0 (0.0) |  |
| 1 answer | 2 (2.4) | 3 (0.9) | 1 (0.2) | 0 (0.0) | 0 (0.0) |  |
| 2 answers | 2 (2.4) | 6 (1.8) | 7 (1.7) | 1 (0.3) | 1 (3.3) |  |
| 3 answers | 5 (6.0) | 10 (3.0) | 14 (3.4) | 7 (2.0) | 2 (6.7) |  |
| 4 answers | 5 (6.0) | 27 (8.2) | 20 (4.8) | 6 (1.7) | 4 (13.3) |  |
| 5 answers | 12 (14.5) | 36 (11.0) | 38 (9.2) | 21 (5.9) | 3 (10.0) |  |
| 6 answers | 14 (16.9) | 50 (15.2) | 53 (12.8) | 43 (12.1) | 3 (10.0) |  |
| 7 answers | 13 (15.7) | 51 (15.5) | 67 (16.1) | 51 (14.3) | 3 (10.0) |  |
| 8 answers | 20 (24.1) | 66 (20.1) | 74 (17.8) | 58 (16.3) | 5 (16.7) |  |
| 9 answers | 1 (1.2) | 39 (11.9) | 70 (16.9) | 71 (19.9) | 6 (20.0) |  |
| 10 answers | 4 (4.8) | 28 (8.5) | 39 (9.4) | 55 (15.4) | 3 (10.0) |  |
| 11 answers | 0 (0.0) | 10 (3.0) | 21 (5.1) | 28 (7.9) | 0 (0.0) |  |
| 12 answers | 2 (2.4) | 1 (0.3) | 9 (2.2) | 9 (2.5) | 0 (0.0) |  |
| 13 answers | 1 (1.2) | 0 (0.0) | 1 (0.2) | 5 (1.4) | 0 (0.0) |  |
|  |  |  |  |  |  |  |
| **Research responses** |  |  |  |  |  |  |
| Participated in prior research |  |  |  |  |  | **<0.001** |
| No | 62 (74.7) | 263 (80.2) | 313 (75.4) | 220 (61.8) | 25 (83.3) |  |
| Yes | 16 (19.3) | 53 (16.2) | 92 (22.2) | 127 (35.7) | 3 (10.0) |  |
| Missing | 5 (6.0) | 12 (3.7) | 10 (2.4) | 9 (2.5) | 2 (6.7) |  |
|  |  |  |  |  |  |  |
| Willing to consider future research survey |  |  |  |  |  | **<0.001** |
| No | 15 (18.1) | 68 (20.7) | 74 (17.8) | 48 (13.5) | 5 (16.7) |  |
| Yes | 43 (51.8) | 185 (56.4) | 274 (66.0) | 272 (76.4) | 14 (46.7) |  |
| Missing | 25 (30.1) | 75 (22.9) | 67 (16.1) | 36 (10.1) | 11 (36.7) |  |
| Willing to consider giving blood for research |  |  |  |  |  | **<0.001** |
| No | 42 (50.6) | 166 (50.6) | 217 (52.3) | 166 (46.6) | 17 (56.7) |  |
| Yes | 16 (19.3) | 87 (26.5) | 131 (31.6) | 154 (43.3) | 2 (6.7) |  |
| Missing | 25 (30.1) | 75 (22.9) | 67 (16.1) | 36 (10.1) | 11 (36.7) |  |
| Willing to consider taking medication for research |  |  |  |  |  | **<0.001** |
| No | 55 (66.3) | 211 (64.3) | 285 (68.7) | 256 (71.9) | 17 (56.7) |  |
| Yes | 3 (3.6) | 42 (12.8) | 63 (15.2) | 64 (18.0) | 2 (6.7) |  |
| Missing | 25 (30.1) | 75 (22.9) | 67 (16.1) | 36 (10.1) | 11 (36.7) |  |
| Willing to consider changing behavior for research |  |  |  |  |  | **<0.001** |
| No | 45 (54.2) | 182 (55.5) | 239 (57.6) | 189 (53.1) | 14 (46.7) |  |
| Yes | 13 (15.7) | 71 (21.6) | 109 (26.3) | 131 (36.8) | 5 (16.7) |  |
| Missing | 25 (30.1) | 75 (22.9) | 67 (16.1) | 36 (10.1) | 11 (36.7) |  |
| Interest in serving as a research advisor |  |  |  |  |  | **<0.001** |
| No | 62 (74.7) | 222 (67.7) | 236 (56.9) | 143 (40.2) | 18 (60.0) |  |
| Maybe | 9 (10.8) | 73 (22.3) | 105 (25.3) | 122 (34.3) | 0 (0.0) |  |
| Yes | 3 (3.6) | 6 (1.8) | 33 (8.0) | 49 (13.8) | 1 (3.3) |  |
| Checked all | 2 (2.4) | 13 (4.0) | 18 (4.3) | 21 (5.9) | 1 (3.3) |  |
| Missing | 7 (8.4) | 14 (4.3) | 23 (5.5) | 21 (5.9) | 10 (33.3) |  |
|  |  |  |  |  |  |  |
| Willing to be contacted by email |  |  |  |  |  | **<0.001** |
| No | 48 (57.8) | 163 (49.7) | 229 (55.2) | 201 (56.5) | 9 (30.0) |  |
| Yes | 8 (9.6) | 54 (16.5) | 95 (22.9) | 108 (30.3) | 2 (6.7) |  |
| Missing | 27 (32.5) | 111 (33.8) | 91 (21.9) | 47 (13.2) | 19 (63.3) |  |
| Willing to be contacted by mailed letter |  |  |  |  |  | **<0.001** |
| No | 27 (32.5) | 110 (33.5) | 143 (34.5) | 142 (39.9) | 10 (33.3) |  |
| Yes | 29 (34.9) | 107 (32.6) | 181 (43.6) | 167 (46.9) | 1 (3.3) |  |
| Missing | 27 (32.5) | 111 (33.8) | 91 (21.9) | 47 (13.2) | 19 (63.3) |  |
| Willing to be contacted by text message |  |  |  |  |  | **<0.001** |
| No | 54 (65.1) | 191 (58.2) | 282 (68.0) | 266 (74.7) | 11 (36.7) |  |
| Yes | 2 (2.4) | 26 (7.9) | 42 (10.1) | 43 (12.1) | 0 (0.0) |  |
| Missing | 27 (32.5) | 111 (33.8) | 91 (21.9) | 47 (13.2) | 19 (63.3) |  |
| Willing to be contacted by recorded phone call |  |  |  |  |  | **<0.001** |
| No | 56 (67.5) | 214 (65.2) | 311 (74.9) | 292 (82.0) | 10 (33.3) |  |
| Yes | 0 (0.0) | 3 (0.9) | 13 (3.1) | 17 (4.8) | 1 (3.3) |  |
| Missing | 27 (32.5) | 111 (33.8) | 91 (21.9) | 47 (13.2) | 19 (63.3) |  |
| Willing to be contacted by live phone call |  |  |  |  |  | **<0.001** |
| No | 53 (63.9) | 183 (55.8) | 265 (63.9) | 244 (68.5) | 11 (36.7) |  |
| Yes | 3 (3.6) | 34 (10.4) | 59 (14.2) | 65 (18.3) | 0 (0.0) |  |
| Missing | 27 (32.5) | 111 (33.8) | 91 (21.9) | 47 (13.2) | 19 (63.3) |  |
| Willing to be contacted by in-person clinic visit |  |  |  |  |  | **<0.001** |
| No | 51 (61.4) | 194 (59.1) | 279 (67.2) | 259 (72.8) | 11 (36.7) |  |
| Yes | 5 (6.0) | 23 (7.0) | 45 (10.8) | 50 (14.0) | 0 (0.0) |  |
| Missing | 27 (32.5) | 111 (33.8) | 91 (21.9) | 47 (13.2) | 19 (63.3) |  |
| Do not contact for future research |  |  |  |  |  | **<0.001** |
| No | 56 (67.5) | 217 (66.2) | 324 (78.1) | 309 (86.8) | 18 (60.0) |  |
| Yes | 27 (32.5) | 111 (33.8) | 91 (21.9) | 47 (13.2) | 12 (40.0) |  |
